# Reasons for Non-adherence to Antiretroviral Therapy Among Adult Patients Receiving free Treatment at a Tertiary Care Hospital in Delhi

**DOI:** 10.4103/0970-0218.62590

**Published:** 2010-01

**Authors:** Vivek Lal, Shashi Kant, Richa Dewan, Sanjay K Rai

**Affiliations:** All India Institute of Medical Sciences, New Delhi, India; 1 Lok Nayak Hospital, New Delhi, India

## Introduction

Adherence to therapies is a primary determinant of treatment success in HIV/AIDS. Poor adherence attenuates optimum clinical benefits and therefore reduces the overall effectiveness of health systems.(1)

It is often said that the most effective regimen for an HIV-infected individual is the one they will take. Both patients and healthcare providers face significant challenges with respect to adherence to antiretroviral therapy (ART). Once initiated, highly active antiretroviral therapy (HAART) is a life-long treatment that consists of multiple medications to be taken two to three times a day with varying dietary instructions. These medications also have side effects, some of which may be temporary, while others may be more permanent requiring a change of treatment.

Unlike other chronic diseases, the rapid replication and mutation rate of HIV means that very high levels of adherence are required to achieve a durable suppression of viral load. Inadequate adherence to treatment is associated with detectable viral loads, declining CD4 counts, disease progression, episodes of opportunistic infections, and poorer health outcomes.(2,3)

India-specific data on adherence is sparse. In the light of the expansion of free ART in the country, there is a need to learn what works and what does not. The reasons for non-adherence to ART need to be studied in order to identify patients who may need support in maintaining adherence and explore the means to do so.

## Materials and Methods

We conducted a cross-sectional, hospital-based study at the ART Clinic of the Lok Nayak Hospital (LNJP) at New Delhi, to ascertain the reasons for non-adherence to ART. LNJP is a tertiary care government hospital. This hospital was identified by the Government of India as one of the first sites in India to offer free ART. The clinic has been functional since April 2004. The clinic provides free ART to patients. The clinic functions as a daily, morning Out-Patient Department (OPD).

The inclusion criteria for the study population included: HIV/AIDS patients attending OPD clinics, on self-administered HAART for at least one week, and of age 18 years or above. The patients who were unable to comprehend the study objectives and process, or who suffered from any acute medical condition, which made the patient unable to participate in the interview or any psychiatric condition due to which the patient could not give a valid consent, were excluded from the study.

The period of data collection was from January 2005 to December 2005. At the end of the study, we were able to enroll 200 patients. Consecutive sampling procedures were adopted and OPD cards were marked, to ensure that each patient was interviewed only once.

The study instrument consisted of a semi-structured anonymous interview schedule. This was prepared in English and translated into Hindi.

Adherence was assessed retrospectively over the previous four-day period, as used in the AIDS Clinical Trials Group (ACTG) follow-up questionnaire.(4) To achieve the highest likelihood of maximal viral suppression, adherence should be 95% or more over time.(5) This meant missing less than one dose for a twice daily regimen. Respondents self-reported when they last missed an antiretroviral (ARV) dose and using a checklist indicated the common reasons as to why they skipped their medication.

Ethical clearance was obtained from the institutional review committee at LNJP prior to the start of the study. A written consent was obtained from each patient prior to enrollment.

Data was entered in Microsoft Excel and was analyzed using SPSS 10.0.

## Results

All the patients who fulfilled the eligibility criteria and were asked to enroll in the study gave their consent.

The number of patients interviewed at LNJP was 200, with a mean age of 33.3 years. The majority of the patients were male. Most of the patients interviewed were between the age group of 31 and 45 years.

It was found that 90% were adherent over the previous four-day period.

A total of 30% of the patients claimed to have ever missed a dose. Multiple responses were allowed for the patients in response to the reasons for ever having missed an ARV dose. The most commonly cited reasons were ‘away from home’ and ‘simply forgot’ [Table 1].

**Table 1:** Reasons for having ever missed an antiretroviral dose

Reasons	Number of responses* (%)
Were away from home	21 (10.5)
Were busy with other things	3 (1.5)
Simply forgot	19 (9.5)
Had too many pills to take	1 (0.5)
Wanted to avoid side effects	3 (1.5)
Did not want others to notice you taking medication	1 (0.5)
Had a change in daily routine	4 (2)
Felt like the drug was toxic/harmful	1 (0.5)
Fell asleep/slept through dose time	2 (1)
Fell sick/ill	13 (6.5)
Felt depressed/overwhelmed	4 (2)
Problem taking pills as specified (enpty stomach/with meal)	Nil
Ran out of pills	3 (1.5)
Felt good	1 (0.5)
Did not have knowledge/was not told of dosing	Nil

* Multiple responses

## Discussion

The study results showed a high level of adherence over the previous four-day period (90%), however, a total of 30% of patients reported having ever missed a dose.

The most common reasons cited for missing doses were ‘away from home’ and ‘simply forgot’. Several other studies have also reported one of the commonest barriers to adherence were absent-mindedness/forgetfulness(6–8) and being away from home.(9,10) Although almost all the patients reported having been told about proper dosing, 6.5% reported having ever missed a dose when they felt sick. This underlines the importance of specifically communicating to the patients the importance of taking the pills as prescribed, even when ill.

The data proves that free ART has been taken up well by the patients and lends support to the decision of the scaling up of free ART. At the same time it puts greater responsibility on the system in terms of sustainability of the free ART program and support through formal counseling sessions, in order to help patients to continue to adhere to this lifelong therapy.

## References

[1] (2003). Adherence to long-term therapies: Evidence for action.

[2] Torres RA, Barr M (1997). Impact of combination therapy for HIV infection on inpatient census. N Engl J Med.

[3] McNaghten AD, Hanson DL, Jones JL, Dworkin MS, Ward JW (1999). Effects of antiretroviral therapy and opportunistic illness primary chemoprophylaxis on survival after AIDS diagnosis. AIDS.

[4] Chesney MA, Ickovics JR, Chambers DB, Gifford AL, Neidig J, Zwickl B (2000). Self-reported adherence to antiretroviral medications among participants in HIV clinical trials: The AACTG adherence instruments: Patient Care Committee and Adherence Working Group of the Outcomes Committee of the Adult AIDS Clinical Trials Group (AACTG). AIDS Care.

[5] Paterson DL, Swindells S, Mohr J, Brester M, Vergis EN, Squier C (2000). Adherence to protease inhibitor therapy and outcomes in patients with HIV infection. Ann Intern Med.

[6] Molassiotis A, Nahas-Lopez V, Chung WY, Lam SW, Li CK, Lau TF (2002). Factors associated with adherence to antiretroviral medication in HIV-infected patients. Int J STD AIDS.

[7] Murphy DA, Roberts KJ, Hoffman D, Molina A, Lu MC (2003). Barriers and successful strategies to antiretroviral adherence among HIV-infected monolingual Spanish-speaking patients. AIDS Care.

[8] Iliyasu Z, Kabir M, Abubakar IS, Babashani M, Zubair ZA (2005). Compliance to antiretroviral therapy among AIDS patients in Aminu Kano Teaching Hospital, Kano, Nigeria. Niger J Med.

[9] Nachega JB, Stein DM, Lehman DA, Hlatshwayo D, Mothopeng R, Chaisson RE (2004). Adherence to antiretroviral therapy in HIV-infected adults in Soweto, South Africa. AIDS Res Hum Retroviruses.

[10] Maggiolo F, Ripamonti D, Arici C, Gregis G, Quinzan G, Camacho GA (2002). Simpler regimens may enhance adherence to antiretrovirals in HIV-infected patients. HIV Clin Trials.

